# Supplementary figures and images for: Zinc Differentially Modulates Tau Aggregation, Fibril Morphology, and Prion-like Seeding in a Construct-Dependent Manner
Source: bioRxiv. 2026 Jul 2:2026.07.01.735859. Preprint. [Version 1] doi: 10.64898/2026.07.01.735859 (PMC13345083; doi:10.64898/2026.07.01.735859)

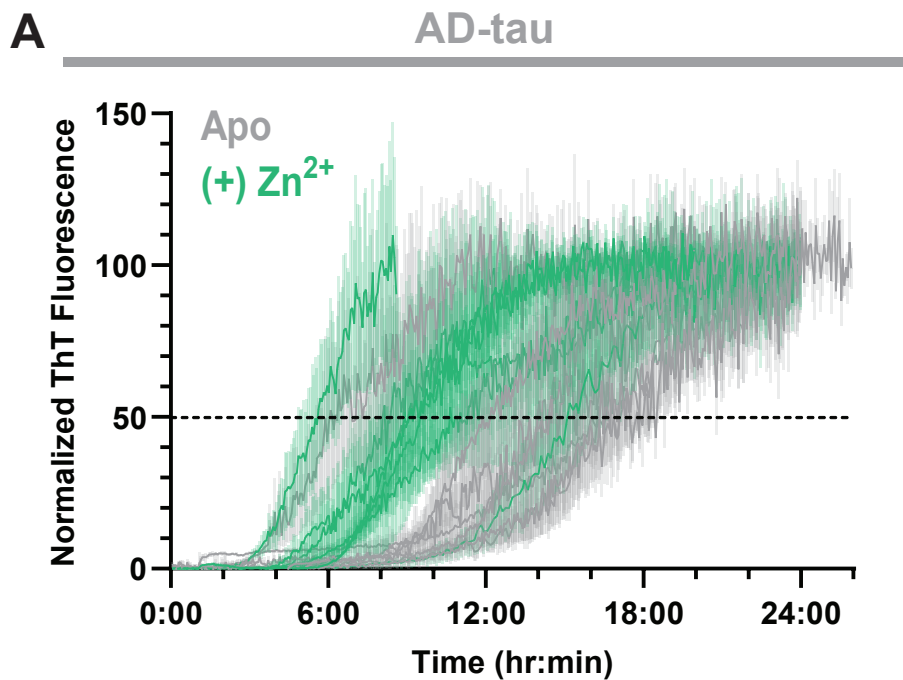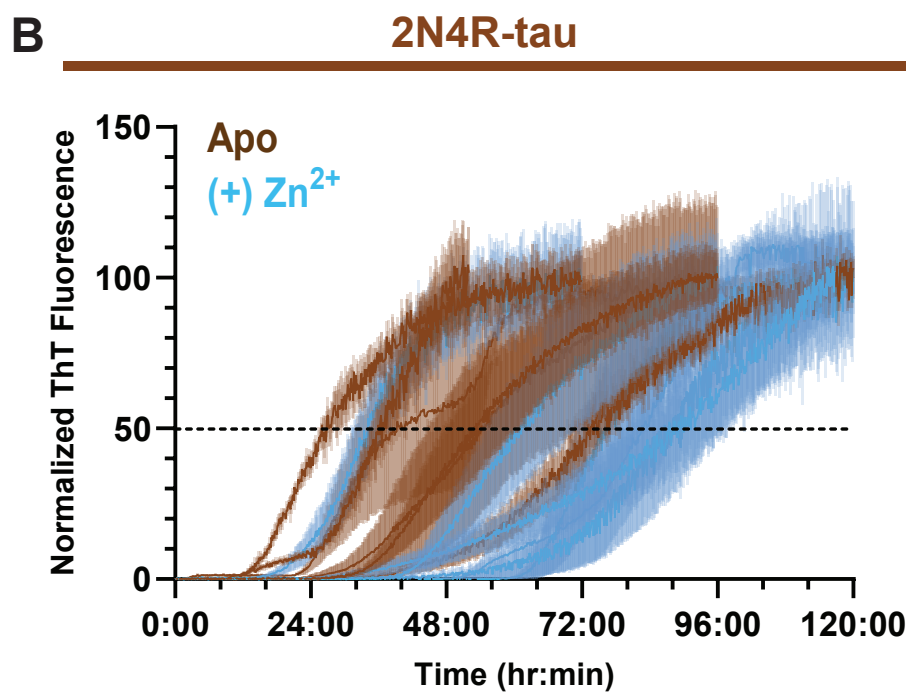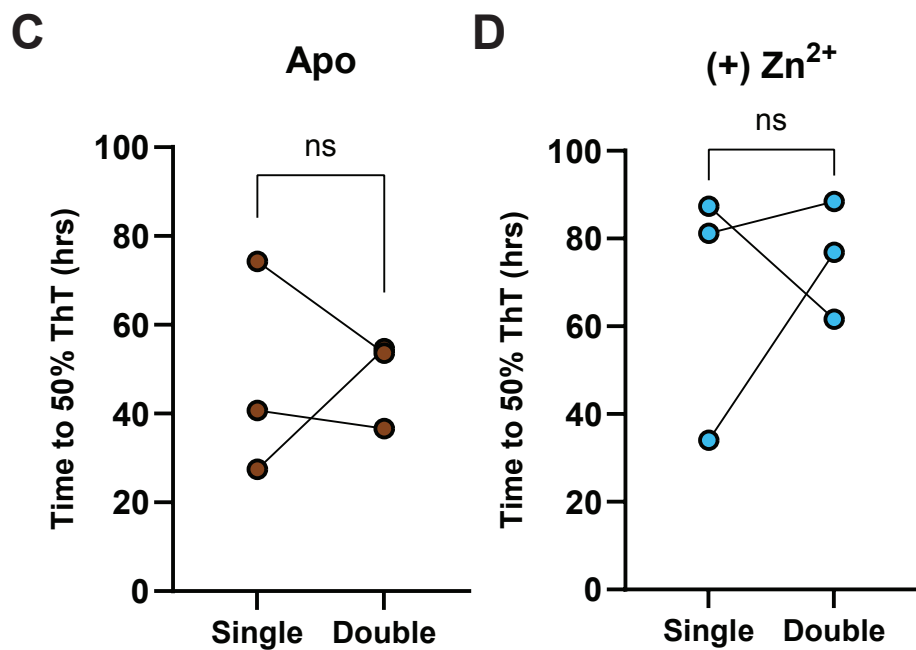

Supplement: Supplement 1 — Supplemental Figure 1. Biological replicates of AD-tau and 2N4R-tau ThT aggregation and comparison of shaking conditions A) ThT aggregation curves for N = 7 AD-tau biological replicates (single orbital shaking), each with n = 3–6 technical replicates. 50 μM tau alone (“Apo,” grey) or with 1.3 mM ZnSO4 (“(+) Zn2+,” green). Each technical replicate normalized individually; mean ± SEM plotted per biological replicate. B) ThT aggregation curves for N = 6 2N4R-tau biological replicates (“Apo,” brown; “(+) Zn2+,” blue). Three assays with single orbital shaking, three with double orbital. C,D) Y50 (hours) compared between single and double orbital shaking for apo (C) and (+) Zn2+ (D). Each shaking type: N = 3 biological replicates with n = 3–6 technical replicates each. Each point represents the mean of one biological replicate. No significant difference between shaking types for either condition (paired t-tests). [file media-1.pdf]

# AD-tau

# 2N4R-tau

**A**

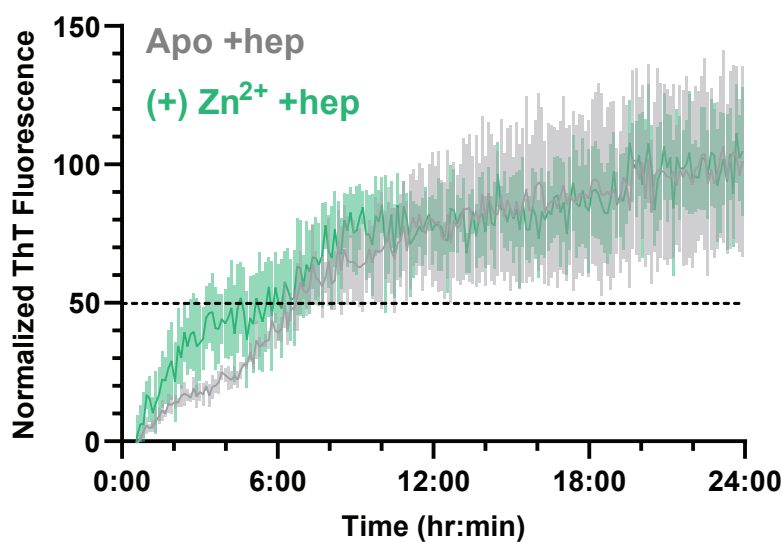

**B**

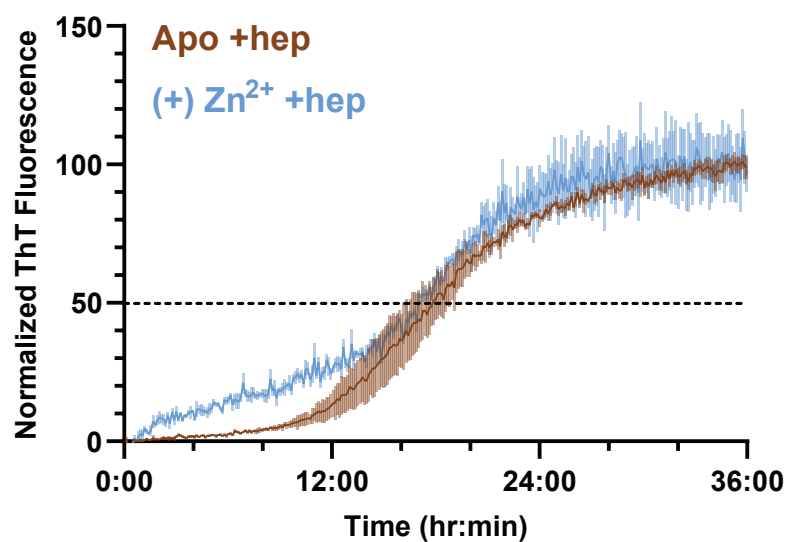

**C**

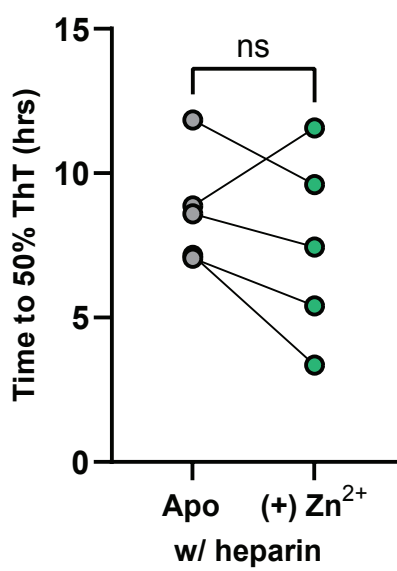

**D**

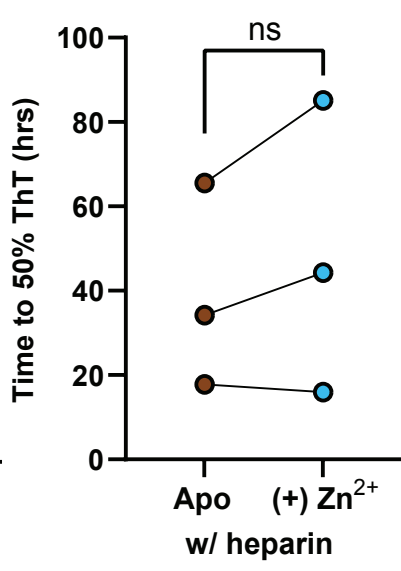

**E**

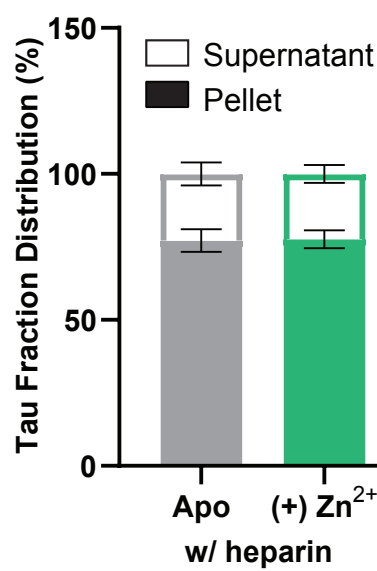

**F**

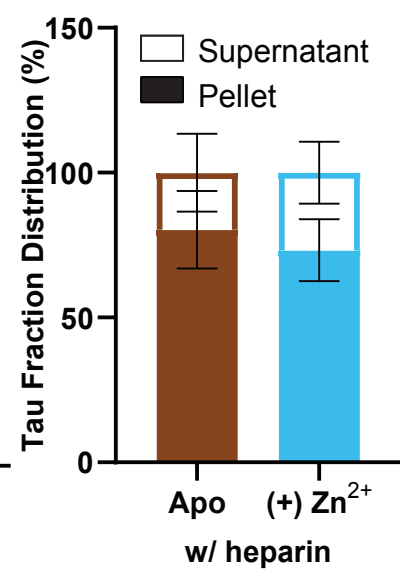

**G**

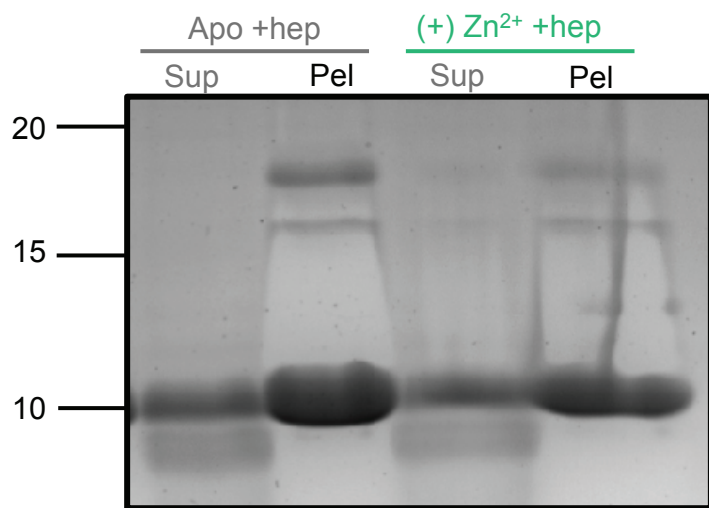

**H**

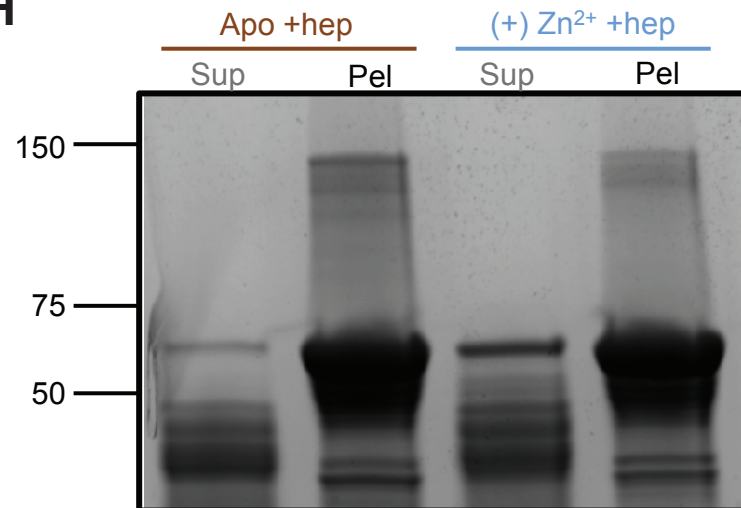

Supplement: Supplement 2 — Supplemental Figure 2. Heparin inclusion masks the effect of Zn2+ on aggregation kinetics and seeding potential A) Representative AD-tau ThT aggregation curve with added heparin, single orbital shaking. 50μM tau + 12.5μM heparin (“Apo +hep,” grey) or 50μM tau + 12.5μM heparin + 1.3 mM ZnSO4 (“(+) Zn2+ +hep,” green). n = 3 technical replicates, each normalized individually; mean ± SEM. B) Representative 2N4R-tau ThT aggregation curve with added heparin, single orbital shaking. 50μM tau + 12.5μM heparin (“Apo +hep,” brown) or 50μM tau + 12.5μM heparin + 1.3 mM ZnSO4 (“(+) Zn2+ +hep,” blue). n = 3 technical replicates, each normalized individually; mean ± SEM. C) Y50 (hours) for AD-tau + heparin. N = 5 biological replicates, each with n ≥ 3 technical replicates. Each point represents the mean of one biological replicate. No significant difference (paired t-test). D) Y50 (hours) for 2N4R-tau + heparin. N = 3 biological replicates, each with n ≥ 3 technical replicates. Each point represents the mean of one biological replicate. No significant difference (paired t-test). E) AD-tau pellet fraction as a percentage of total tau (Sup + Pel), quantified by densitometry, from 3 biological replicates. Mean ± SEM plotted. No significant difference using a paired t-test. F) 2N4R-tau pellet fraction as a percentage of total tau (Sup + Pel), quantified by densitometry, from 2 biological replicates. Mean ± SEM plotted. No significant difference using a paired t-test. G) Representative SDS-PAGE of supernatant (Sup) and pellet (Pel) fractions after ultracentrifugation of AD-tau aggregation. H) Representative SDS-PAGE of supernatant (Sup) and pellet (Pel) fractions after ultracentrifugation of 2N4R-tau aggregation. [file media-2.pdf]

## AD-tau

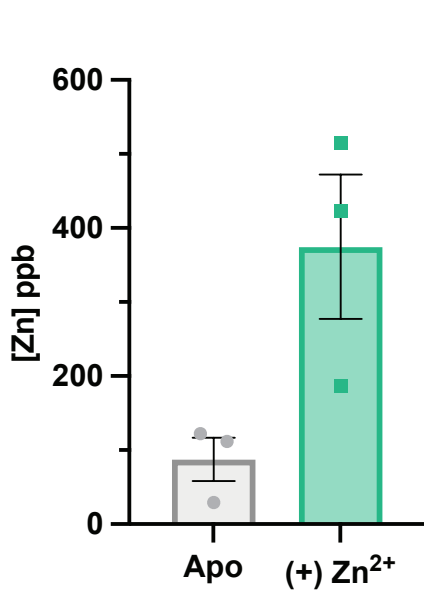

## 2N4R-tau

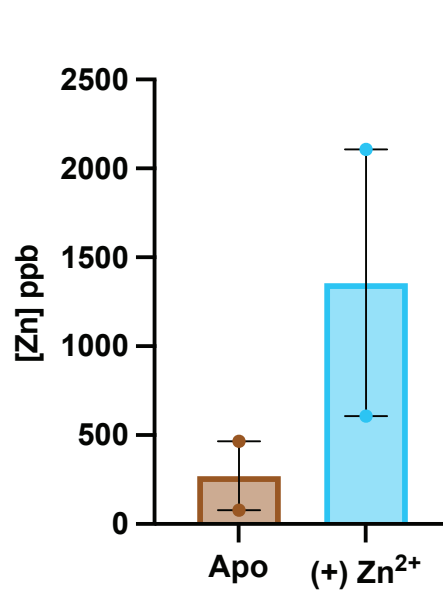

Supplement: Supplement 3 — Supplemental Figure 3: Zinc is incorporated into both AD-tau and 2N4R-tau fibrils A) Zinc (ppb) per μmol AD-tau fibrils, measured by ICP-MS. N = 3 biological replicates; mean ± SEM. B) Zinc (ppb) per μmol 2N4R-tau fibrils, measured by ICP-MS. N = 2 biological replicates; mean ± SEM. [file media-3.pdf]
