# Supplementary material for: Zinc Differentially Modulates Tau Aggregation, Fibril Morphology, and Prion-like Seeding in a Construct-Dependent Manner
Source: bioRxiv. 2026 Jul 2:2026.07.01.735859. Preprint. [Version 1] doi: 10.64898/2026.07.01.735859 (PMC13345083; doi:10.64898/2026.07.01.735859)
Supplement: Supplement 4 — Supplemental Figure 4. Flow cytometry gating scheme and independent trials of the cellular seeding assay A) Gating scheme for flow cytometry analysis of the FRET seeding assay. No-seed and seeded “FRET positive” gates are shown to illustrate the difference between conditions. B) Integrated FRET density for 4 independent trials of AD-tau seeding. Conditions: no seed (black), Zn2+ alone (purple), apo (grey), apo + Zn2+ post-aggregation (dark grey), (+) Zn2+ (green). Each trial: n = 3 biological replicates (total N = 12); mean ± SEM. *p < 0.0332, **p < 0.0021, ***p < 0.0002, ****p < 0.0001 (one-way ANOVA with Tukey’s post hoc test). C) Integrated FRET density for 3 independent trials of 2N4R-tau seeding. Conditions: no seed (black), Zn2+ alone (purple), apo (brown), apo + Zn2+ post-aggregation (dark brown), (+) Zn2+ (blue). Each trial: n = 3 biological replicates (total N = 9); mean ± SEM. *p < 0.0332, **p < 0.0021, ***p < 0.0002, ****p < 0.0001 (one-way ANOVA with Tukey’s post hoc test). [file media-4.pdf]

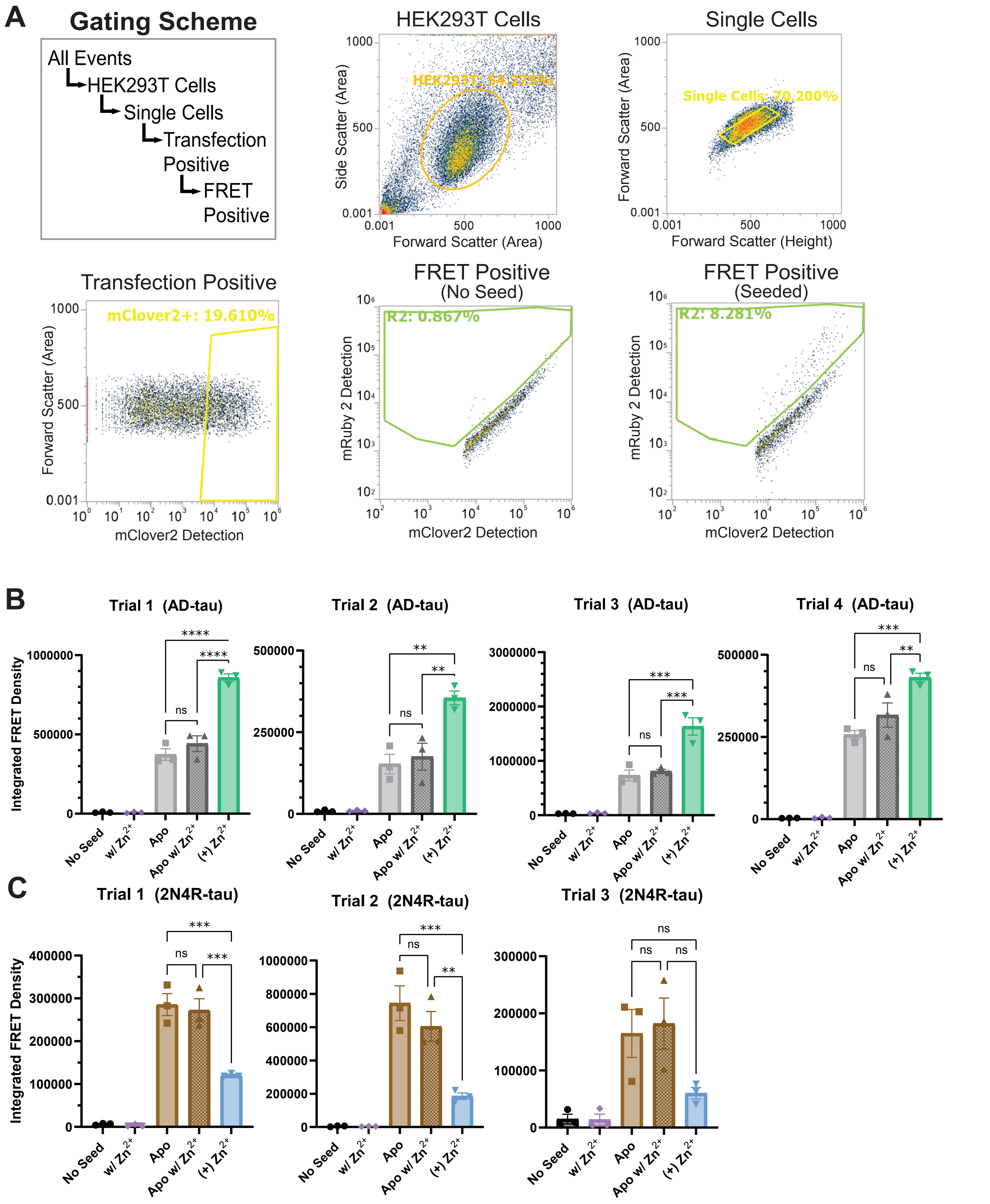

A
Gating Scheme
All Events
HEK293T Cells
Single Cells
Transfection Positive
FRET Positive
HEK293T Cells
HEK293T: 54.275%
Side Scatter (Area)
Forward Scatter (Area)
Single Cells
Single Cells: 70.200%
Forward Scatter (Area)
Forward Scatter (Height)
Transfection Positive
mClover2+: 19.610%
Forward Scatter (Area)
mClover2 Detection
FRET Positive (No Seed)
R2: 0.867%
mRuby 2 Detection
mClover2 Detection
FRET Positive (Seeded)
R2: 8.281%
mRuby 2 Detection
mClover2 Detection
B
Trial 1 (AD-tau)
Trial 2 (AD-tau)
Trial 3 (AD-tau)
Trial 4 (AD-tau)
Integrated FRET Density
No Seed
w/ Zn2+
Apo
Apo w/ Zn2+
(+) Zn2+
C
Trial 1 (2N4R-tau)
Trial 2 (2N4R-tau)
Trial 3 (2N4R-tau)
Integrated FRET Density
No Seed
w/ Zn2+
Apo
Apo w/ Zn2+
(+) Zn2+
